# Lignin Removal in Subcellular Location of Poplar Cell Wall During Pretreatment Significantly Impacts Cellulose Digestibility

**DOI:** 10.1021/acssuschemeng.5c00692

**Published:** 2025-05-20

**Authors:** Jian Zhang, Lin Kang, Wei Shen, Cynthia Collings, Heng Gong, Kirk vander Meulen, Brian G. Fox, Elise Gilcher, James A. Dumesic, Shi-You Ding

**Affiliations:** † School of Biotechnology, 47860 East China University of Science and Technology, 130 Meilong Road, Shanghai 200237, China; ‡ Department of Plant Biology, 3078 Michigan State University, East Lansing, Michigan 48824, United States; § DOE Great Lakes Bioenergy Research Center, Michigan State University, East Lansing, Michigan 48824, United States; ∥ Xiamen Key Laboratory of Rare Earth Photoelectric Functional Materials, Xiamen Institute of Rare Earth Materials, Haixi Institute, Chinese Academy of Sciences, 258 Duishan Road, Xiamen, Fujian 361021, China; ⊥ Fujian Institute of Research on the Structure of Matter, Chinese Academy of Sciences, 155 Yangqiao Road West, Fuzhou, Fujian 350002, China; # DOE Great Lakes Bioenergy Research Center, Madison, Wisconsin 53706, United States; ¶ Department of Biochemistry, 5228 University of Wisconsin-Madison, Madison, Wisconsin 53706, United States; ∇ Department of Chemical and Biological Engineering, University of Wisconsin-Madison, Madison, Wisconsin 53706, United States

**Keywords:** GVL–HCl pretreatment, lignin removal, subcellular location, CBM–GFP binding, stimulated Raman scattering microscopy (SRS), enzyme digestibility

## Abstract

The γ-valerolactone (GVL) pretreatment is one of the leading solvent-based methods for producing high-quality lignin under mild conditions. However, the glucan conversion yield from GVL pretreated biomass remains unsatisfactory. To explore the discrepancies between the relatively low glucan conversion and high lignin extraction, we conducted GVL–HCl and NaOH pretreatments on poplar and investigated their effects on lignin content and location, as well as on enzymatic hydrolysis of poplar cell walls at the subcellular level. Under designated pretreatment conditions of GVL–HCl (90% GVL, 0.1 M HCl, 100 °C, 1 h) and NaOH (1 M, 121 °C, 2 h), the glucan conversion yields were 69.4% and 95.8%, with lignin removal rates of 67.8% and 47.7%, respectively. Four types of GFP-labeled carbohydrate binding modules were used to identify different forms of cellulose in the pretreated cell walls. The overall binding intensities to pretreated poplar were stronger for NaOH compared to GVL–HCl pretreatment. Stimulated Raman scattering microscopy imaging revealed that GVL–HCl preferentially extracted lignin from the compound middle lamella and cell corner areas, while NaOH effectively dissolved lignin in the secondary cell walls. Real-time imaging of cellulase degradation of pretreated cell walls further indicated that digestion started from both the cell lumen and the compound middle lamella areas for GVL, whereas it occurred uniformly across the secondary cell walls for NaOH. Our findings suggest that the location of lignin removal during pretreatment is crucial for enzymatic cellulose degradation, in addition to the total amount of lignin extraction.

## Graphical abstract

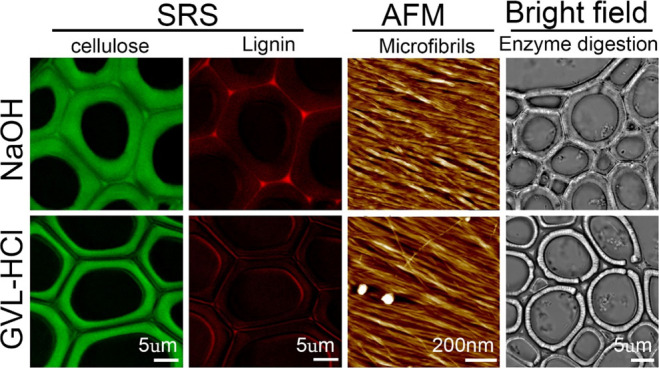

## 1 Introduction

Lignocellulosic biomass is the most abundant renewable resource in the world. Cellulose-based biofuels and biochemicals have great potential to replace fossil-based products. The hydrophobic lignin cross-links hemicelluloses forming a matrix and then coats the surface of cellulose microfibrils, which are heterogeneously distributed in plant cell walls, inhibiting the access of glucan to cellulases.^1^ Therefore, pretreatment is the first critical step in the biochemical conversion process of lignocellulose, designed to overcome plant cell wall recalcitrance and improve sugar yield during subsequent enzymatic hydrolysis.^2^ Numerous pretreatment methods have been developed to dissolve lignin, not only to enhance the fermentable sugar yields, but also to upgrade the extracted lignin into high-value aromatic products, making the overall biorefinery process economically feasible.^3,4^

γ-Valerolactone (GVL) pretreatment is one of the leading solvent-based methods, well-known for its high-quality lignin extraction under mild conditions.^5^ GVL-extracted lignin preserves more β-*O*-4 ether bonds and aliphatic hydroxyl groups, facilitating the subsequent lignin valorization.^6,7^ However, the glucan conversion yield of GVL-pretreated biomass remains unsatisfactory (<70%) compared to the high lignin extraction rate (>50%). For instance, Shuai et al. dissolved 81.8% of lignin from hardwood during GVL pretreatment (80% GVL, 75 mM H_2_SO_4_, 120 °C for 2 h), yet the glucose yield after enzymatic hydrolysis was only 63%.^5^ Zhou et al. reported a glucose yield of 48% with ∼50% lignin removal under a condition of 80% GVL, 0.1 M H_2_SO_4_, 120 °C for 1 h.^8^ Jia et al. achieved a glucan conversion ratio of approximately 60% from GVL pretreated corn stover (80% GVL, 75 mM H_2_SO_4_) with 75.4% lignin removal.^9^ Hu et al. obtained a glucose yield of 67.0% with 55.3% delignification rate after GVL pretreatment (70% GVL, 0.1 M HCl, 120 °C for 20 min) of corn stover.^4^ Similarly, in our recent study,^10^ the glucose yield reached 65% with 68% lignin removal using GVL pretreated poplar (90% GVL, 0.1 M HCl, 100 °C for 1 h).

Other pretreatment methods have also reported the phenomenon that a higher amount of lignin removal does not necessarily result in a higher sugar yield. Li et al. obtained a sugar yield of 45.67% from deep eutectic solvent pretreated *pinus* (choline chloride/lactic acid = 1:10, in molar ratio, 120 °C for 4 h) with 66.59% lignin removal.^11^ Some hypotheses, such as GVL grafting onto cellulose via esterification during pretreatment or excessive lignin removal causing cellulose structure collapse, as well as accumulation of more recalcitrant form of cellulose Iβ, have been proposed to explain the lower glucan conversion.^3,5,12^

In addition to the amount of lignin removal, which is usually measured via bulk analysis at a macro-scale and reflects the average lignin content from the entire plant cell wall, the location of the lignin removal during pretreatment has been suggested as another important factor influencing enzymatic hydrolysis.^13,14^ However, few studies have comprehensively investigated the relationship between the spatial distribution of lignin removal in plant cell walls during pretreatment and cellulose conversion in the subsequent enzymatic hydrolysis.

In this study, besides GVL–HCl pretreatment, another pretreatment method well-known for lignin removal, NaOH pretreatment were conducted to examine their effects on lignin removal in terms of both content and subcellular locations, as well as on enzymatic hydrolysis efficiency. First, the chemical compositions of pretreated poplar biomass and glucose yield after enzyme hydrolysis were analyzed. Four types of GFP–labeled carbohydrate binding modules (CBMs–GFP) were used to specifically bind to different parts of the cell wall and confocal laser scanning microscopy was employed to characterize microstructure changes after the pretreatments. Additionally, the distribution of both carbohydrates and lignin in poplar cell walls was visualized using stimulated Raman scattering microscopy (SRS). Atomic force microscopy (AFM) was also performed to assess the impact of lignin removal on microfibrils structures. Finally, enzyme hydrolysis was observed under light microscopy to visualize in real-time the morphological changes in pretreated cell walls. The findings from this study provide fundamental insights into the relationship between lignin removal at the subcellular level during pretreatment and glucose yield after enzyme hydrolysis. These insights will be valuable for upgrading both GVL-based pretreatment and other lignin removal pretreatment methods in the future.

## 2 Material and Methods

### 2.1 Biomass Materials

A 25–30 y old hybrid poplar Populus nigra var. charkoviensis *x* caudina cv. NE-19 harvested in Arlington, Wisconsin, USA, was provided by Great Lakes Bioenergy Research Center (GLBRC, Madison, Wisconsin). For imaging experiments, transverse and longitudinal sections of poplar were prepared by hand-cutting using a single-blade razor. The slices were checked with bright-field light microscopy to select samples with relatively uniform cuttings and approximately 25 μm in thickness. For enzymatic hydrolysis tests, the debarked poplar chips were ground into less than 5 mm particles using a Wiley mill before pretreatment.

### 2.2 GVL–HCl Pretreatment and NaOH Pretreatment

GVL–HCl pretreatment was conducted as described previously.^10^ The ground poplar particles were added in a 100 mL glass flask to a 10% solids loading with 40 mL of GVL/H_2_O (90:10, in volume) and 0.1 M HCl. Then the flask was heated to 100 °C in a boiling water bath for 60 min. After pretreatment, the solid was washed with 500 mL deionized water to remove solvents, and then dried in an oven at 40 °C overnight. The composition of the solid poplar was analyzed according to National Renewable Energy Laboratory (NREL) standard protocols.^15^

NaOH pretreatment was conducted at a 10% solid loading (weight ratio) using ground poplar particles (4.0 g) in a 250 mL glass flask with 1 M NaOH solution. The flasks were put into the autoclave and heated to 121 °C for 2 h. The solid was washed with 500 mL deionized water to a neutral pH, and then dried in an oven at 40 °C overnight. The composition of the solids was analyzed before use in enzymatic hydrolysis.

### 2.3 Confocal Laser Scanning Microscopy

A confocal laser scanning microscope (Leica DMi8 microscope equipped with Crestoptics X-light V2 confocal system) was used for imaging the binding of GFP-tagged CBMs to plant cell walls. GFP was excited by a 473 nm laser and coupled with a 525 nm emission filter.

For monitoring, 5 μL of the CBM–GFP proteins (50 μg/mL) were added to a 1.5 mL plastic tube containing 20 μL citric acid buffer then incubated with the poplar slice for 30 min at room temperature. After that, the poplar slice was washed with deionized water three times before being monitored by CLSM. The preparations of *Tr*CBM1-GFP and *Ct*CBM3-GFP proteins were described in Ding et al.^16^ The preparations of *Rt*CBM6-GFP and *Rt*CBM44-GFP proteins were described in Walker et al.^17^ All images were recorded at the resolution of 1024 × 1024 pixels, and were analyzed using ImageJ (http://rsb.info.nih.gov/ij).

### 2.4 Two-Color Coherent Raman Scattering (SRS) Microscopy

SRS microscopy was performed on the samples using a two-color instrument same as the one described previously.^18^ Briefly, the spectral focusing hyperspectral SRS imaging method was used for fast hyperspectral scanning. SRS imaging was performed using a dual-output laser system (InSight DeepSee, Spectra-Physics) with ultrafast excitation sources. Two broadband lasers were spatiotemporally synchronized within an inverted laser-scanning microscope (Olympus IX83, equipped with a Fluoview 1200 scanning head), emitting pulses of 1.5 ps at an 80 MHz repetition rate. The 1040 nm laser, serving as the Stokes beam, was modulated by an electro-optical modulator (EOM) at 20 MHz, while a tunable pump laser was directed through a motorized delay stage, adjusted to 797 nm for the 2900 cm^–1^ band and 889 nm for the 1600 cm^–1^ band, enabling the visualization of polysaccharides and lignin, respectively. For detection, the Stokes beam was blocked using a Chroma short-pass filter (ET890/220m), while the SRS signal, corresponding to stimulated Raman loss of the pump beam after passing through the sample, was captured by a photodiode and analyzed using a lock-in amplifier (APE GmbH) at 20 MHz.

### 2.5 Atomic Force Microscopy Operation

The cellulose microfibers of longitudinal and transverse slices of poplar were monitored at room temperature on a Dimension AFM with Nanoscope controller V (Fastscan, Bruker Nano, Santa Barbara, CA, USA) with an acoustic and vibration isolation system. Probes used were SCANASYST-Air (Bruker, Camarillo, CA, USA) for imaging in the air. The AFM operation software (Nanoscope V9.1) was used to control the scan size, setpoint, and gain. Before imaging, the scanner was calibrated using a calibration kit (Bruker, Camiarillo, CA, USA). All images were obtained at a scan rate of 2 Hz with a resolution of 512 × 512 pixels.^19^

### 2.6 Enzymatic Hydrolysis

The enzymatic hydrolysis of the pretreated poplar solid was conducted in a vial containing 10 mL 0.05 M citric acid buffer (pH 4.8). The glucan loading was 1% (weight base) with Cellic CTec3 (Novozymes, Franklinton, NC, USA) dosage of 15 mg protein/g glucan. The reaction lasted for 96 h at 50 °C, 250 rpm in a shaking incubator. The samples taken after enzymatic hydrolysis were analyzed on HPLC. Every hydrolysis experiment was conducted in triplicate.

The monitoring of cell wall digestion by cellulases was carried out in a sealed chamber (SLF-0201, Bio-Rad, Hercules, CA, USA). One piece of pretreated poplar slice was rinsed three times with citric acid buffer (0.05 M, pH 4.8) before digestion. The digestion system contained 30 μL diluted Cellic CTec3 with a protein concentration of 15 mg/mL. Enzymatic reactions were carried out at room temperature for 96 h. The images were taken every hour with a resolution of 1024 × 1024 pixels using a bright-field light microscopy.

### 2.7 HPLC Analysis

Glucose, xylose, furfural and HMF were determined using an Agilent Infinity 1260 HPLC fitted with a Refractive Index Detector and Bio-Rad Aminex HPX-87H column operated at 55 °C using 5 mM H_2_SO_4_ as eluent at a rate of 0.6 mL/min.

## 3 Results

### 3.1 Chemical Composition and Glucan Digestibility of NaOH and GVL–HCl Pretreated Poplar

The NaOH pretreatment conditions used here (1 M NaOH, 121 °C for 2 h) were adapted from Ji et al. (2014), and the severity was increased slightly.^13^ While the GVL–HCl pretreatment conditions (90% GVL with 0.1 M HCl, 100 °C for 1 h) were optimized in our previous study.^10^ As shown in Figure 1, the chemical composition of the pretreated solids revealed that the glucan content increased to 52.3% following NaOH pretreatment and to 69.6% with GVL–HCl pretreatment, primarily due to the extraction of lignin and xylan into the pretreatment liquid by the solvents. Based on solids recovery (51.4% for NaOH pretreatment, and 48.9% for GVL–HCl pretreatment) and chemical composition of the pretreated solids, the lignin removal ratio was calculated as 47.7% by NaOH pretreatment, and 67.8% by GVL–HCl pretreatment, compared with the untreated poplar.

**Figure 1:**
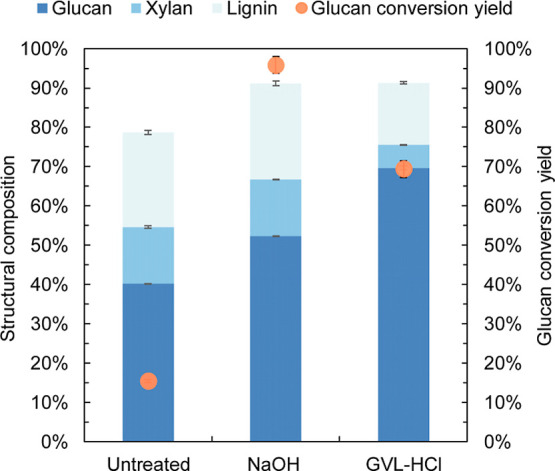
Chemical composition of untreated and pretreated (either NaOH or GVL–HCl) poplar and glucan conversion yield after 96 h enzymatic hydrolysis. The composition of the pretreated poplar was based on the dry solids recovered. The standard deviations were calculated based on the results of experiments conducted in triplicate.

Enzymatic hydrolysis of the pretreated poplar solids was conducted using Cellic Ctec3 at a dosage of 15 mg protein/g glucan for 96 h. Unexpectedly, GVL–HCl pretreated poplar achieved a glucan conversion yield of only 69.4%, significantly lower than the yield of 95.8% obtained from NaOH pretreated poplar. A hypothesis was proposed that esterification might graft GVL onto the cellulose surface during GVL pretreatment, potentially inhibiting glucan conversion.^3,5^ To investigate the discrepancy between the relative low glucan conversion and the high lignin removal ratio in the GVL–HCL pretreatment, multiscale visualization techniques were employed to characterize microstructural changes in the poplar cell wall, alongside the bulk lignin measurements.

### 3.2 Structural Changes in Poplar Cell Wall Characterized by Carbohydrate Binding Modules Binding

Carbohydrate binding modules (CBMs) could specifically bind to polysaccharides and assist in targeting the catalytic domains of glycoside hydrolases to their appropriate carbohydrate substrates.^17,20^ In this study, CBMs fused to GFP (CBMs–GFP) were used to monitor ultrastructure changes of the plant cell wall during pretreatment. Four distinct CBMs–GFP were cloned and purified. *Tr*CBM1, derived from Trichoderma reesei cellobiohydrolase I (CBH I or Cel7A) and *Ct*CBM3, derived from Clostridium thermocellum cellulosomal scaffoldin protein (CipA), specifically recognize the planar face of crystalline cellulose, facilitating the hydrolysis of crystalline cellulose.^15^ *Rt*CBM6, cloned from a Ruminoclostridium thermocellum xylanase, primarily binds to xylan.^21,22^ *Rt*CBM44, derived from R. thermocellum, has a binding site comprised of a narrow groove lined with hydrophobic aromatic residues and specifically detects amorphous regions of cellulose.^22^ CBM–GFP binding to the poplar cell wall was visualized using CLSM, with excitation at 473 nm and observed emission at 525 nm.

The bright field images in Figure 2a show that the poplar cell wall remained intact but became slightly thicker due to the swelling effect of NaOH pretreatment. After GLV–HCl pretreatment, the compound middle lamella and cell corners appeared almost void, and part of the primary cell wall was peeled away from the secondary cell wall. The average cell wall thickness increased by approximately 60% following NaOH pretreatment, while GVL–HCl pretreatment had minimal effect on cell wall thickness. In Figure 2b, *Tr*CBM1-GFP bound evenly to the surface of secondary poplar cell walls, with minor differences in binding intensity between the two pretreatments. As shown in Figure 2c, *Ct*CBM3-GFP demonstrated a binding preference for both the inner (near the cell lumen) and outer surfaces (near the cell corner and compound middle lamella) of the GVL–HCl pretreated poplar cell wall, whereas the binding was uniform throughout the entire secondary cell walls in NaOH pretreated poplar. Given that *Ct*CBM3 has a weaker penetration capacity than *Tr*CBM1, its uniform binding pattern indicates that NaOH pretreatment makes cellulose in the secondary cell walls more accessible to cellulases. For *Rt*CBM6-GFP binding in Figure 2d, the weaker binding observed in the GVL–HCl pretreated solids is likely due to reduced xylan content compared to NaOH pretreated samples. The binding intensity of *Rt*CBM44-GFP in Figure 2e was stronger for NaOH treated poplar than for GVL–HCl treated samples, suggesting that NaOH pretreatment increased the amount of amorphous cellulose.

**Figure 2:**
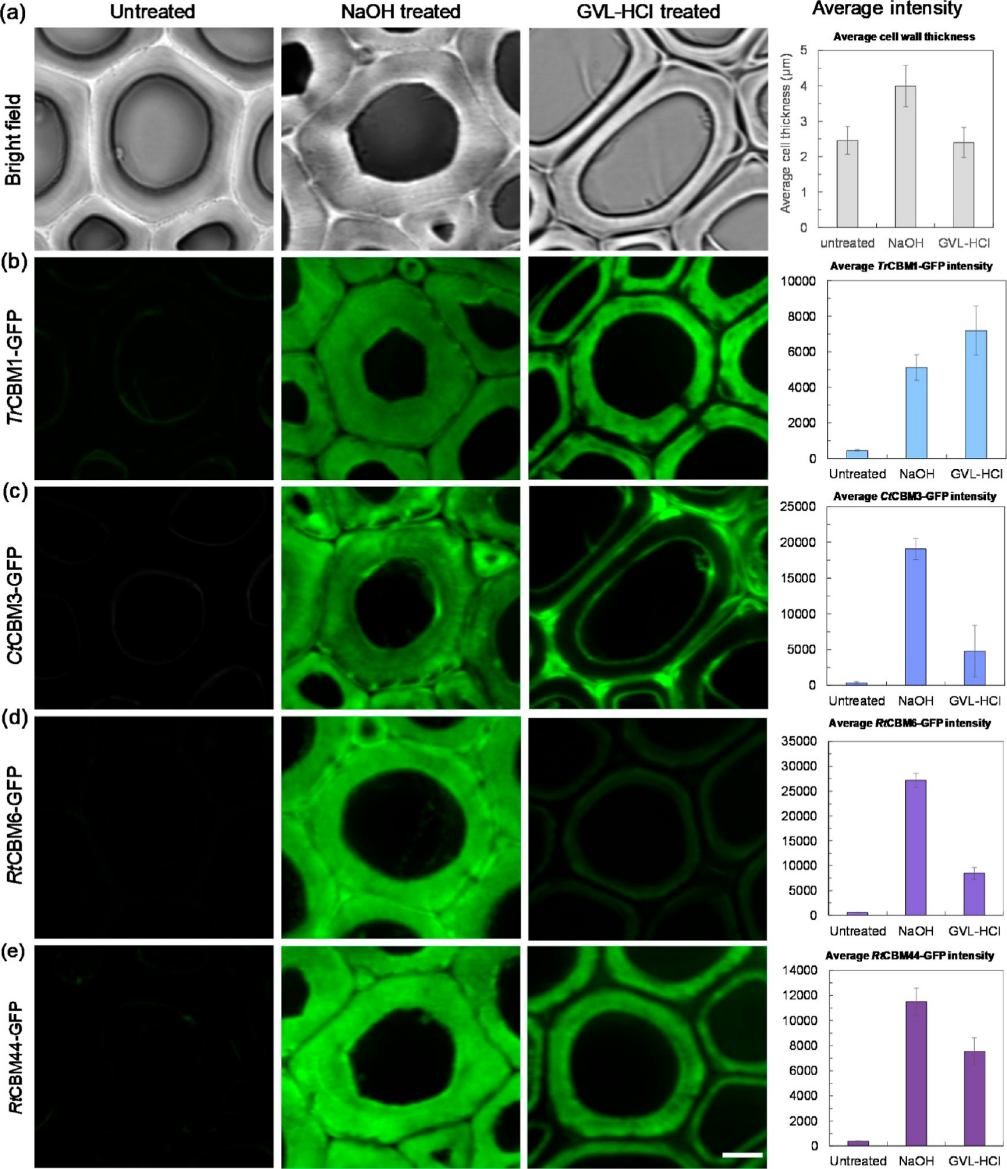
CLSM images of untreated, NaOH–, and GVL–HCl-pretreated poplar cell walls labeled with various CBM–GFP. (a) Bright field images, (b) *Tr*CBM1-GFP, (c) *Ct*CBM3-GFP, (d) *Rt*CBM6-GFP, (e) *Rt*CBM44-GFP. The GFP signal (green) represents the CBM accessibility to either cellulose or xylan in the cell walls before and after NaOH or GVL–HCl pretreatment. Scale bar is 5 μm. The standard deviations of fluorescence intensity were analyzed based on 50 CLSM images.

Overall, the above results indicate that the crystalline and amorphous cellulose, as well as xylan in the poplar cell wall, were more accessible to their respective CBMs following NaOH pretreatment (which showed lower lignin removal) than after GVL–HCL pretreatment. These findings align well with previous glucan digestion results.

### 3.3 Stimulated Raman Scattering Imaging to Spatially Locate Lignin Removal in Plant Cell Wall during Pretreatment

To investigate the spatial distribution of lignin removal in the poplar cell wall, two-color stimulated Raman scattering (SRS) microscopy was used to visualize the distribution of carbohydrates and lignin during pretreatment (Figure 3). We used the Raman signal at 2900 cm^–1^ to indicate polysaccharides and the 1600 cm^–1^ band to indicate lignin.^16,23^ Figure 3a–d show that the solid consisted primarily of polysaccharides after pretreatment. Notably, GVL–HCl removed over 90% of lignin from the compound middle lamella and cell corner areas (Figure 3e–h), consistent with the CBM binding results. More cellulose was accessible to *Ct*CBM3 due to lignin removal from these areas, despite its limited permeability. In contrast, NaOH pretreatment primarily removed lignin from the secondary cell wall, leaving enough lignin to maintain cell adhesion. These SRS results clearly indicate a spatial preference for delignification among the two pretreatments.

**Figure 3:**
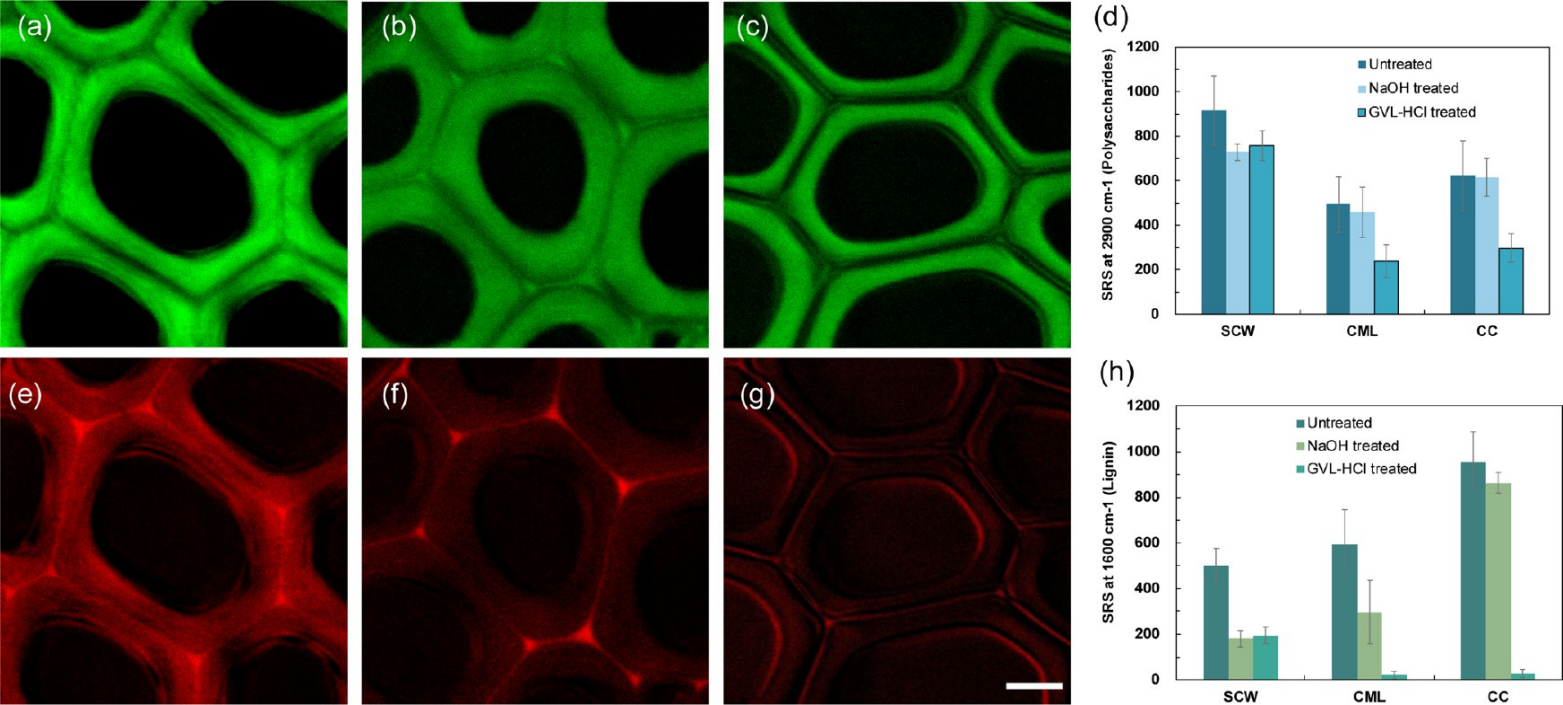
Stimulated Raman scattering (SRS) microscopy of poplar cell walls. Polysaccharide (2900 cm^–1^) fraction of untreated poplar cell walls (a), NaOH-treated poplar cell walls (b), GVL–HCl treated poplar cell walls (c), semiquantitative analysis of SRS signals at 2900 cm^–1^ of different cell wall locations before and after pretreatment (d), and lignin (1600 cm^–1^) fraction of untreated poplar cell walls (e), NaOH-treated poplar cell walls (f), GVL–HCl treated poplar cell walls (g), semiquantitative analysis of SRS signals at 1600 cm^–1^ of different cell wall locations before and after pretreatment (h). Scale bar is 5 μm. The color bar represents the relative fluorescence intensity. The standard deviations of fluorescence intensity were analyzed based on 50 SRS images. SCW: secondary cell wall, CML: compound middle lamella, CC: cell corner.

### 3.4 Atomic Force Microscopy Imaging and Real-Time Visualization of Cell Wall Digestion

AFM images in Figure 4 reveal that matrix-like substances, such as lignin and hemicellulose, which were closely associated with the microfibril surface in the untreated poplar cell wall, became nearly undetectable after both NaOH and GVL–HCl pretreatments. Following NaOH pretreatment, the microfibrils swelled, and individual microfibrils separated from the microfibril bundles (Figure 4b), whereas the microfibril bundles remained intact in the GVL–HCl pretreated poplar (Figure 4c).

**Figure 4:**
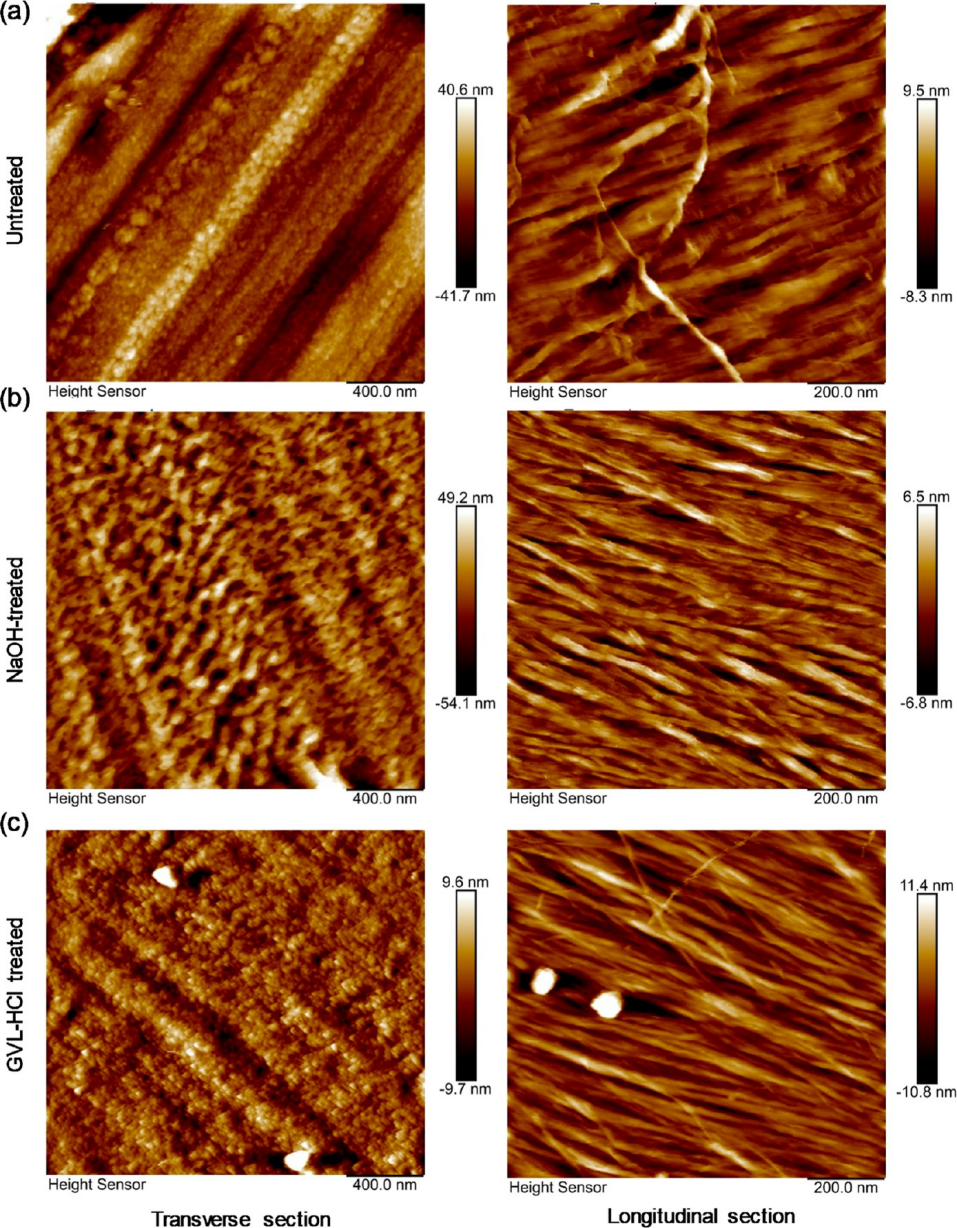
Atomic force micrograph of poplar cell walls before and after pretreatment. (a) Untreated, (b) NaOH-treated, and (c) GVL–HCl treated.

To observe the effect of lignin removal on cellulose digestion, enzymatic hydrolysis of a pretreated poplar slice was monitored for 96 h at room temperature using bright field microscopy. As shown in Figure 5a, NaOH pretreated poplar was digested evenly across the entire secondary cell wall surface. The swelled and separated microfibrils (shown in Figure 4) after NaOH pretreatment exposed more cellulose to cellulases, and were easily to be digested. After 96 h of hydrolysis, achieving a cellulose conversion of 96%, cell wall debris, mainly lignin remnants in the cell corner and middle lamella areas, could still be observed. In Figure 5b, the GVL–HCl pretreated poplar showed digestion beginning in the compound middle lamella area, causing the cell walls to separate from these regions. The secondary cell walls were subsequently digested progressively from both the compound middle lamella and cell lumen sides. These observations align well with the more lignin removal and stronger binding of *Ct*CBM3-GFP to the compound middle lamella and cell lumen areas, as well as better enzyme hydrolysis as shown in Figure 1.

**Figure 5:**
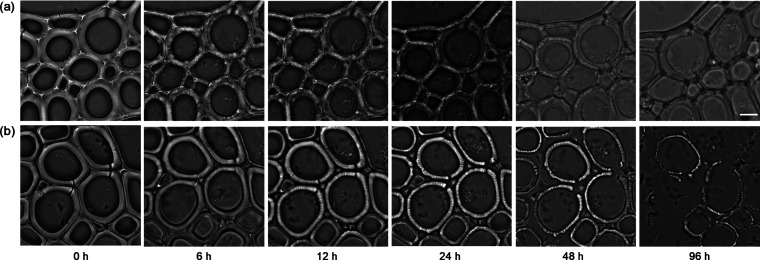
Real-time imaging of the enzymatic hydrolysis of cell walls pretreated by NaOH (a) and GVL–HCl (b). The incubation time lasted for 96 h. Scale bar is 5 μm.

Taken together, these results demonstrate that cellulose digestion begins in subcellular regions with lower lignin content, though the cellulose conversion yield does not directly correlate with the lignin removal ratio. The location of lignin removal during pretreatments is also a key factor influencing cellulose digestibility.

## 4 Discussion

Alkaline and GVL-based pretreatment are both commonly used for delignification, but their selectivity in lignin removal differs. Alkaline pretreatment, i.e., NaOH in this study, targets the secondary cell wall, which contains a high proportion of syringyl lignin with a relative low-density structure.^13,24,25^ In contrast, GVL–HCl pretreatment tends to dissolve lignin in the cell corner and compound middle lamella of the poplar cell walls, areas enriched with condensed guaiacyl lignin.^26,27^ Studies have reported that the molecular weight of lignin extracted by GVL is around 2.5 kDa, significantly larger than the lignin extracted by other solvents. This indicates that GVL is more effective at cleaving intermolecular linkages between lignin and xylan, resulting in large lignin molecules.^3,4,7,28^ While some studies suggested that GVL had a favorable interaction with syringyl lignin. It was important to note that variations in lignocellulosic biomass and pretreatment conditions can lead to differing results.^8,29^

GVL solvent has a high capacity for lignin dissolution; however, most lignin removed is from the cell corner and compound middle lamella areas, which are low in cellulose.^27^ A substantial amount of lignin, which obstructs cellulose accessibility to cellulases and reduces cellulose digestibility, retains after GVL pretreatment in the cellulose-rich secondary cell wall. In contrast, lignin removed by NaOH pretreatment is mainly from the secondary cell wall, significantly enhancing cellulose digestibility by clearing these steric obstructions. Several studies have shown that an alkali post-treatment of GVL pretreated biomass can improve cellulose digestibility from less than 70% to over 95%. It can be inferred that lignin removal from the secondary cell wall during alkali post-treatment is likely responsible for the marked increase in glucan conversion.^5,9^

Efforts to develop and optimize pretreatment methods have always focused on reducing total lignin content, regardless of the specific location of lignin removal.^2^ However, findings in this study demonstrate that cellulose digestibility depends not only on the quantity of lignin removed, but also on the specific subcellular location of lignin removal. Removing lignin from the secondary cell wall is more beneficial for facilitating subsequent cellulose hydrolysis than removing lignin from cell corner and compound middle lamella areas.

## 5 Conclusions

GVL–HCl pretreatment achieved a relatively low glucan conversion yield despite high lignin removal, compared to NaOH pretreatment. Imaging studies showed weaker CBMs–GFP binding intensities to the GVL–HCl pretreated poplar cell wall than to NaOH pretreated cell wall. We further demonstrated that lignin removal at subcellular locations during GVL–HCl pretreatment was primarily from the compound middle lamella and cell corners. In contrast, NaOH pretreatment preferentially dissolved lignin in the secondary cell wall, which significantly enhanced subsequent cellulose hydrolysis. Our study suggests that the specific location of lignin removal during pretreatment is also a critical factor influencing cellulose conversion, in addition to the total amount of lignin extracted.

## Data Availability

The data that support the findings of this study are available from the corresponding author upon reasonable request.
